# A nomogram for predicting subsequent liver metastasis in patients with metastatic breast cancer

**DOI:** 10.3389/fonc.2025.1417858

**Published:** 2025-04-16

**Authors:** Xuanchen Liu, Weipeng Zhao, Yongsheng Jia, Li Zhang, Zhongsheng Tong

**Affiliations:** ^1^ Department of Breast Oncology, Tianjin Medical University Cancer Institute & Hospital, National Clinical Research Center for Cancer, Key Laboratory of Cancer Prevention and Therapy, Tianjin, China; ^2^ Tianjin’s Clinical Research Center for Cancer, Key Laboratory of Breast Cancer Prevention and Therapy, Tianjin Medical University, Ministry of Education, Tianjin, China

**Keywords:** liver metastasis, metastatic breast cancer, nomogram, the fine-gray competing risk model, risk prediction

## Abstract

**Background:**

To investigate the clinical characteristics of liver metastasis from metastatic breast cancer and construct a competing risk nomogram for predicting the probability of liver metastasis.

**Methods:**

Clinical data of patients with metastatic breast cancer from Tianjin Medical University Cancer Institute during 2008–2018 were retrospectively collected. Independent prognostic factors were assessed by the Fine-Gray competing risk model. A competing risk nomogram was constructed by integrating those independent prognostic factors and evaluated with concordance index (C-index) and calibration curves.

**Results:**

A total of 1406 patients were retrospectively analyzed, and randomly divided into the training set (n=986) and the validation set (n=420). Multivariate analysis showed that menopausal status, HER-2 status, bone metastasis and lung metastasis were identified as independent prognostic factors in the nomogram. The C-index in the training set was 0.719 (95% CI: 0.706–0.732), and in the validation set was 0.740 (95% CI: 0.720–0.732). The calibration curves in the training set and validation set showed that the nomogram had a sufficient level of calibration. A risk stratification was further established to divide all the patients into three prognostic groups.

**Conclusion:**

We had developed a tool that can predict subsequent liver metastasis from metastatic breast cancer, which may be useful for identifying the patients at risk of liver metastasis and guiding the individualized treatment. It had been verified that the nomogram has good discrimination and calibration, and had certain potential clinical value. This nomogram can be used to screen patients with low, intermediate and high risk of liver metastasis from metastatic breast cancer, so as to develop a more complete follow-up plan.

## 1 Introduction

As a serious danger to human health, breast cancer is currently a leading cause of cancer mortality worldwide, with 20% of the patients relapsing (1–3). The 5-year survival rate of metastatic breast cancer is only 24% (4, 5). The most frequent sites of metastatic progression of breast cancer are the bone, lung, liver, pleura and soft tissue, among which the liver is the third most common site of distant metastasis (6–8). The incidence of liver metastasis from metastatic breast cancer was 6%–25%, while in IV stage, the incidence was 50% (9). Compared with other sites of metastasis, the liver metastasis from metastatic breast cancer has a worse prognosis because of the significant burden of tumor cell proliferation as well as the gradual deterioration of liver function. Moreover, the liver metastasis is generally asymptomatic and easily overlooked. If it was overlooked, the survival period was typically 4–8 months (10–12). In fact, all patients with advanced breast cancer were at the risk of liver metastasis, so it is particularly important to construct a tool for identifying the timing of liver metastasis and prepare clinical strategies in advance to improve the overall survival of patients.

In recent years, as an indispensable tool for predicting outcomes, nomogram provides individualized risk estimates by incorporating and illustrating important prognostic factors, and it is more accurate in predicting the outcome of cancer patients than other available decision-making aids (13–15). A small number of studies constructed nomograms to predict the liver metastasis from metastatic breast cancer, but none of these studies accounted appropriately for the competing risks (16, 17). The competing risk model refers to that in the observation cohort, if a known event may affect the probability of the occurrence of another event or completely hinder its occurrence, the former and the latter can be considered to have competing risks (18). For example, The occurrence of death will hinder the occurrence of liver metastasis. It is inappropriate to apply Kaplan-Meier and Cox methods in the presence of competing risk.

The purpose of this study is to construct a nomogram based on competing risk model to predict the subsequent liver metastasis from metastatic breast cancer.

## 2 Materials and methods

### 2.1 Data source and study population

This retrospective study was based on a cohort of metastatic breast cancer patients who received first-line treatment at Tianjin Medical University Cancer Institute during 2008–2018. All patients were confirmed to have distant metastasis by histological or radiological examination. Inclusion criteria: 1. Relatively complete clinical data: age, stage at initial diagnosis, menstrual status, site of metastasis, treatment plan, etc. 2. Complete follow-up data. Exclusion criteria: 1. Patients with liver metastasis at the initial metastasis diagnosis 2. The interval between the initial diagnosis and the confirmed diagnosis of liver metastasis was less than 6 months 3. Patients with insufficient (<2 months) follow-up times 4. Patients with double primary cancers.

### 2.2 Study variables

In order to construct this nomogram, we collected 22 clinicopathological factors that may be associated with subsequent liver metastasis from metastatic breast cancer. All information was obtained through electronic medical record system, outpatient follow-up and telephone inquiry, including (1) demographic data: age at initial diagnosis and menstrual status. (2) pathological data: histological subtype, histological grade, T stage, N stage, molecular type, ki-67 index and stage at initial diagnosis of cancer. (3) treatment data: initial surgery. (4) laboratory data: total bilirubin, ALT, AST and lactate dehydrogenase (LDH). (5) past medical history: fatty liver, liver cyst and HBV infection. (6) metastasis characteristics: initial site of metastasis.

In this study, HER-2 expression was assessed using immunohistochemistry (IHC) and fluorescence *in situ* hybridization (FISH). HER-2 positivity was defined as an IHC score of 3+, indicating strong membrane staining in more than 30% of tumor cells, or an IHC score of 2+ confirmed by FISH showing HER-2 gene amplification greater than 2.0. HER-2 negativity was defined as an IHC score of 0 or 1+, with 0 indicating no staining or weak staining, and 1+ indicating that most cells were negative, along with FISH results showing HER-2 gene amplification less than 2.0.

The initial metastatic site and subsequent liver metastasis were determined by pathological biopsy and radiological report. The radiological report included computerized tomography (CT) and magnetic resonance imaging (MRI) for chest, abdomen, pelvis, axillary, head and bone.

Laboratory data were stratified according to clinical cut-off values and were measured within the 2 weeks preceding the first-line treatment of metastatic disease. The cut-off values of total bilirubin, ALT, AST and LDH were based on other studies (18, 19).

### 2.3 Primary endpoint

The primary endpoint was liver metastasis-free survival, which was defined as the time from the first metastasis to the confirmed diagnosis of liver metastasis, and death before liver metastasis was considered to be a competing event (20, 21). Patients without liver metastasis or death were censored at the last follow-up. The last follow-up was conducted on 1 July 2021. If a patient was lost to follow-up, such patient would be censored on the last day of follow-up.

### 2.4 Statistical analysis

The death and the liver metastasis from advanced breast cancer were considered as two competing events. The Fine-Gray competing risk model was used to assess the independent prognostic factors for the subsequent liver metastasis from metastatic breast cancer. Prognostic factors with the *P* value of < 0.05 in the univariate analysis were included into the multivariate analysis. Cumulative incidence function curve was used to assess the probability of each event and the differences between groups were estimated using the Fine-Gray’s test. A nomogram was constructed to visualize this predictive model. The predictive performance of the nomogram was evaluated by discrimination ability and calibration ability. The discrimination ability was evaluated by C-index and the calibration ability was evaluated by calibration curve. Both C-index and calibration curve were calculated by the bootstrap method with 1000 resamples.

A risk stratification was performed on the basis of each patient’s total scores in the nomogram. All the patients were divided into three groups through X-tile software. The cumulative incidence of liver metastasis in different groups was estimated through the cumulative incidence function, taking into consideration the competing risk of death before liver metastasis. Gray’s test was used to compare the differences between groups.

All statistical analyses were performed using the R software version 4.1.1 with the R packages rms, cmprsk, and mstate. All statistical tests were two-tailed and *P* < 0.05 was considered statistically significant.

## 3 Results

### 3.1 Clinicopathologic characteristics of patients

The clinical data of 1406 patients with metastatic breast cancer were retrospectively analyzed. The median follow-up time was 69 (interquartile range 2–197) months from the time of confirmed diagnosis of metastatic disease. The baselines of the training set and the validation set were balanced, where were calculated by the chi-square test (*P*>0.05). Among these patients, 628 patients were younger than 50 years old, and 604 were premenopause. The invasive ductal carcinoma (84.50%) was the most common histological subtype and histological grade II disease (86.49%). The most common initial site of metastasis was non-regional lymph nodes (45.95%), followed by bone (41.68%), lung (31.15%). 300 (21.34%) patients developed liver metastasis, and 215 (15.29%) patients died before liver metastasis. 851 (60.53%) patients had no liver metastasis or death at the end of the last follow-up (**Supplementary Table S1**).

### 3.2 Independent prognostic factors

**Figure 1:**
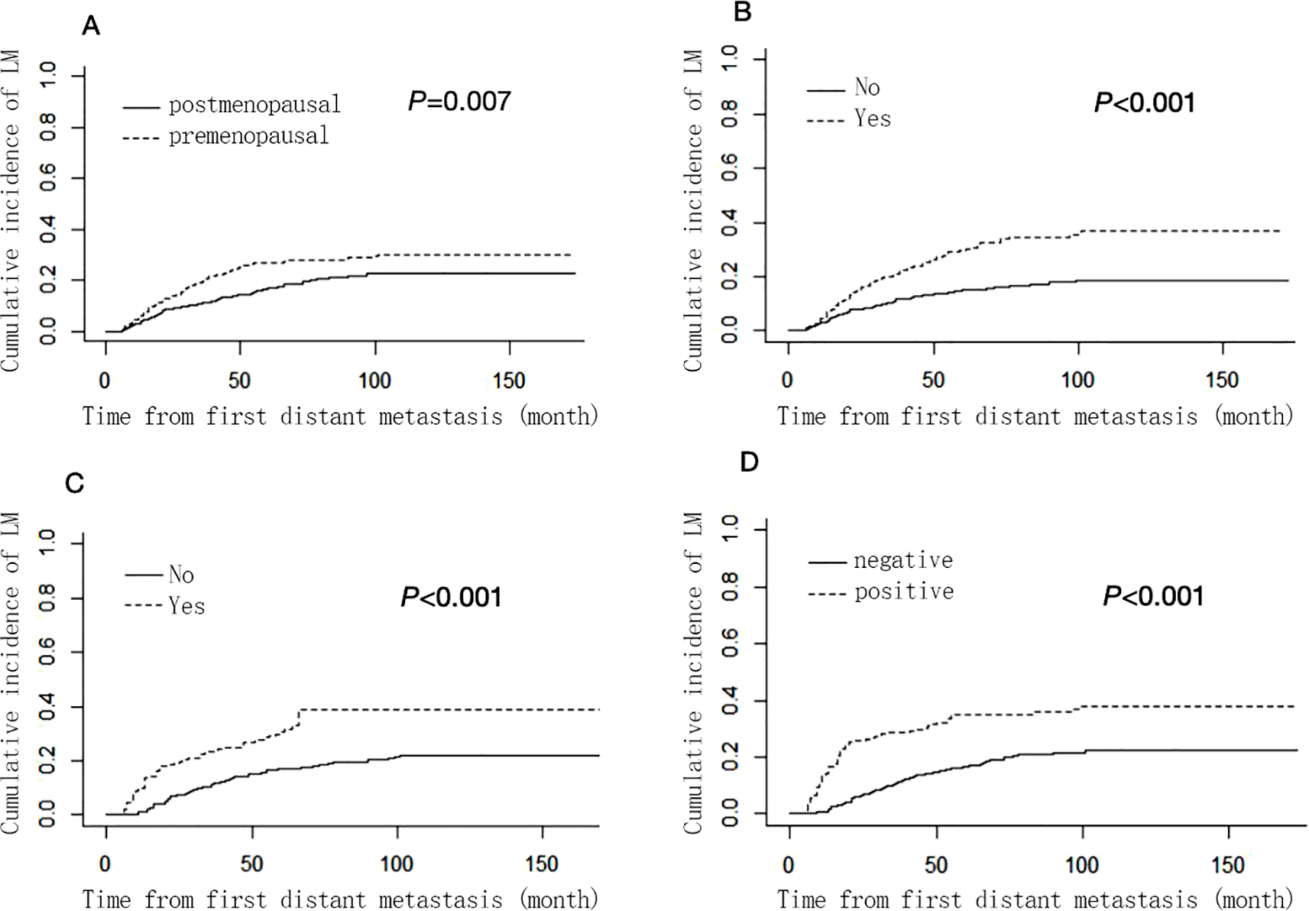
Cumulative liver ,metastasis incidence curves stratified by menopausal status **(A)**, bone metastasis **(B)**, lung metastasis **(C)** and HER-2 status **(D)**. .

**Supplementary Table S2** shows the results of univariable analysis and multivariable analysis based on the Fine-Gray competing risk model. Menopausal status (premenopausal: SHR 1.52, 95% CI: 1.12–2.06, postmenopausal as a reference), HER-2 status (positive: SHR 2.29, 95% CI: 1.71–3.03, negative as a reference), lung metastasis (metastasis: SHR 2.01, 95% CI: 1.53–2.65, no metastasis as a reference) and bone metastasis (metastasis: SHR 1.89, 95% CI: 1.43–2.50, no metastasis as a reference) remained as independent prognostic factors for liver metastasis and were used to construct nomogram. However, the hormone receptors ER and PR did not show statistically significant differences and were therefore not included in the nomogram. The cumulative incidence function curves of these independent prognostic factors are shown in **Figure 1**.

### 3.3 Nomogram construction and evaluation

**Figure 2:**
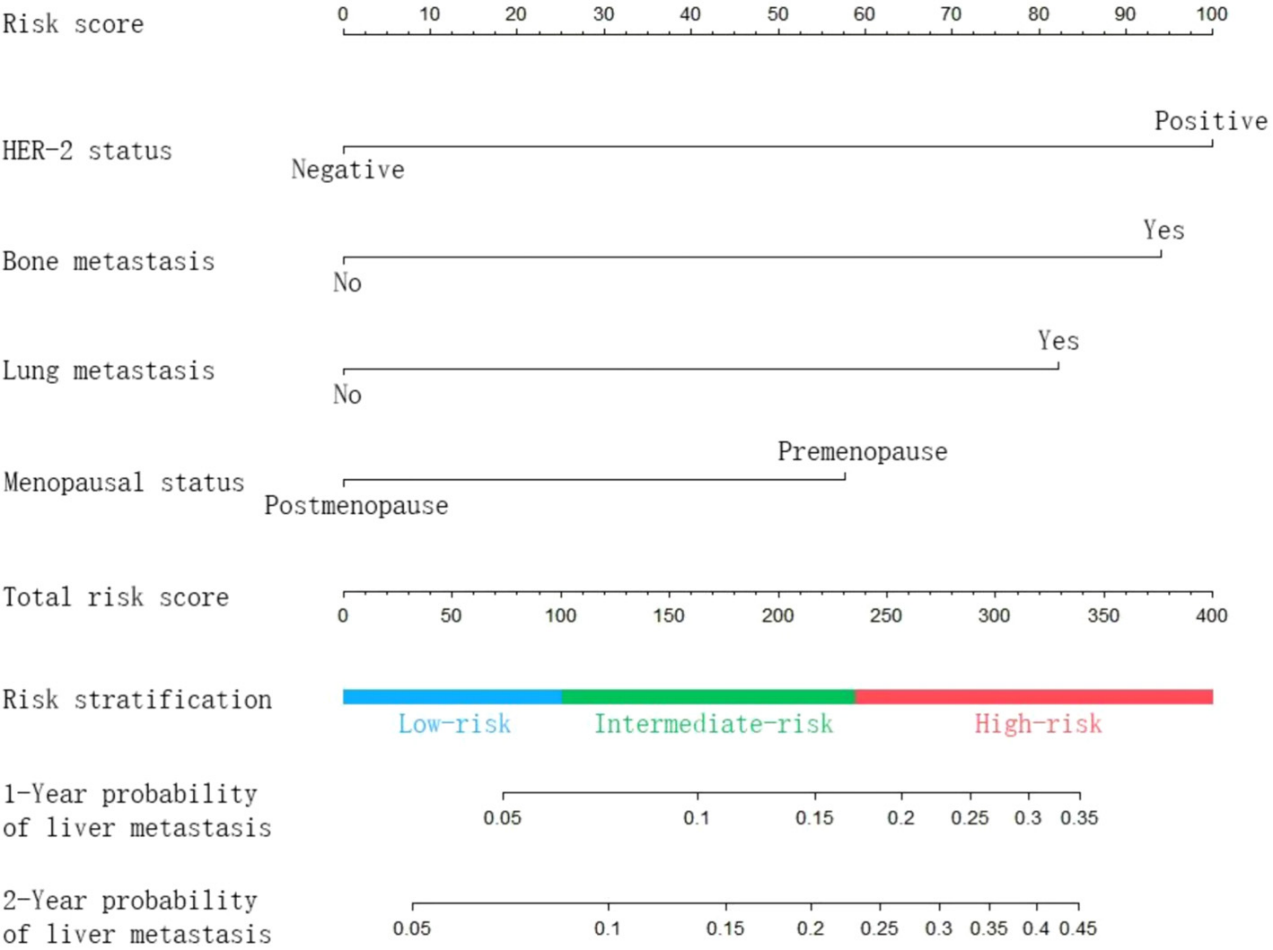
Nomogram predicting the probability of subsequent liver metastasis in patients with metastatic breast cancer.

The nomogram (**Figure 2**) was established based on four independent risk factors. The corresponding scores of each clinical factor are shown in **Supplementary Table S3**. The HER-2 status (negative: 0 points, positive: 100 points) and bone metastasis (metastasis: 94 points, no metastasis: 0 points) contributed the most to the development of liver metastasis. Besides, lung metastasis (metastasis: 82 points, no metastasis: 0 points) and menopausal status (premenopausal 58 points, postmenopausal: 0 points) showed a moderate effect on the development of liver metastasis. By adding the corresponding scores of each factor, the probability of liver metastasis at a specific time point can be obtained.

**Figure 3:**
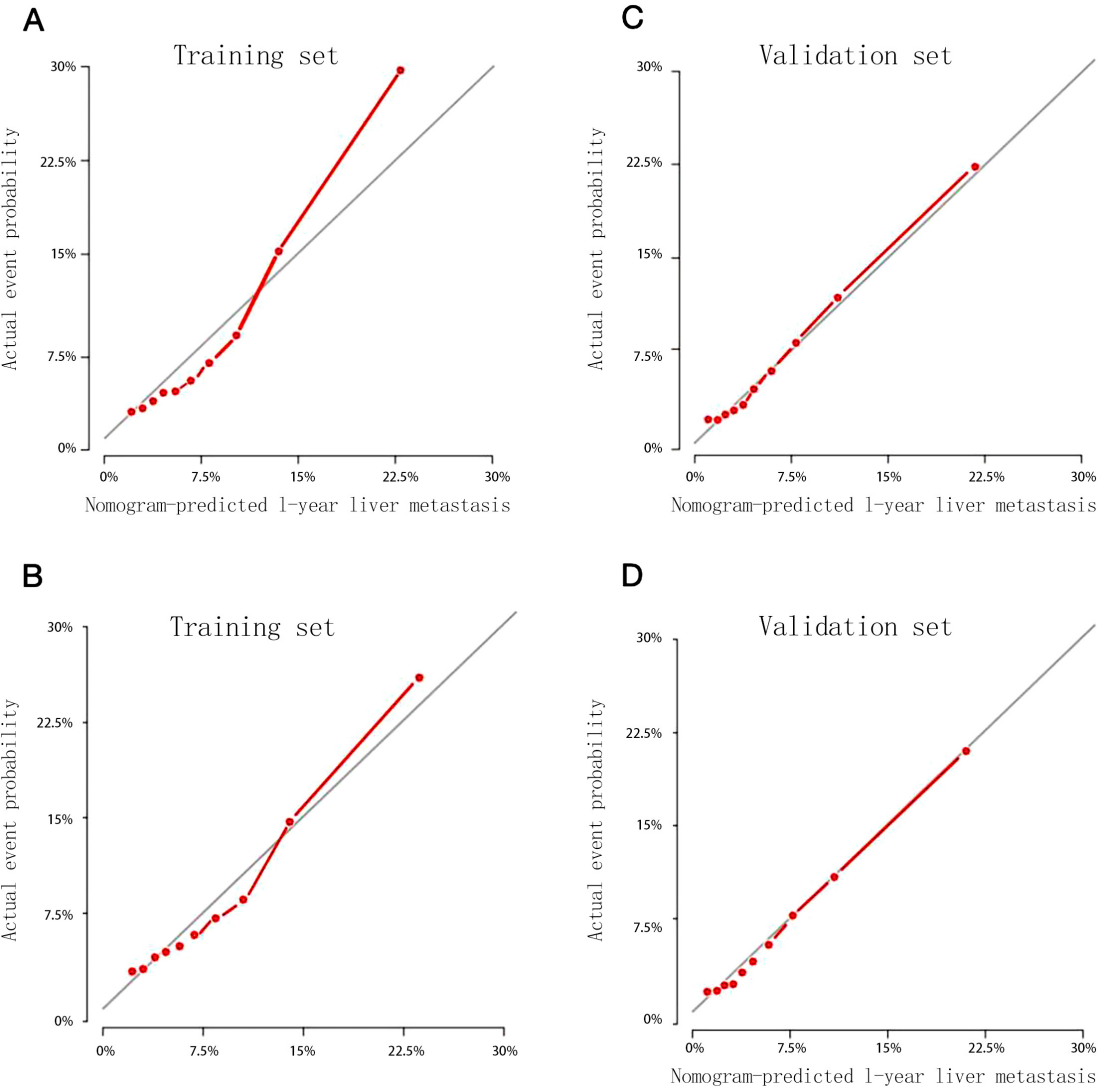
Calibration curves for predicting 1-year **(A)** and 2-year **(B)** probability of liver metastasis in the training set, 1-year **(C)** and 2-year **(D)** probability of liver metastasis in the validation set.

Being calculated by bootstrap method with 1000 resamples, the C-index in the training set was 0.719 (95% CI: 0.706–0.732), and in the validation set was 0.740 (95% CI: 0.720–0.760). The 12-month and 24-month calibration curves of the training set and validation set showed that the nomogram-predicted probability and the actual probability of occurrence were highly consistent (**Figure 3**).

### 3.4 Establishment of risk stratification system

**Figure 4:**
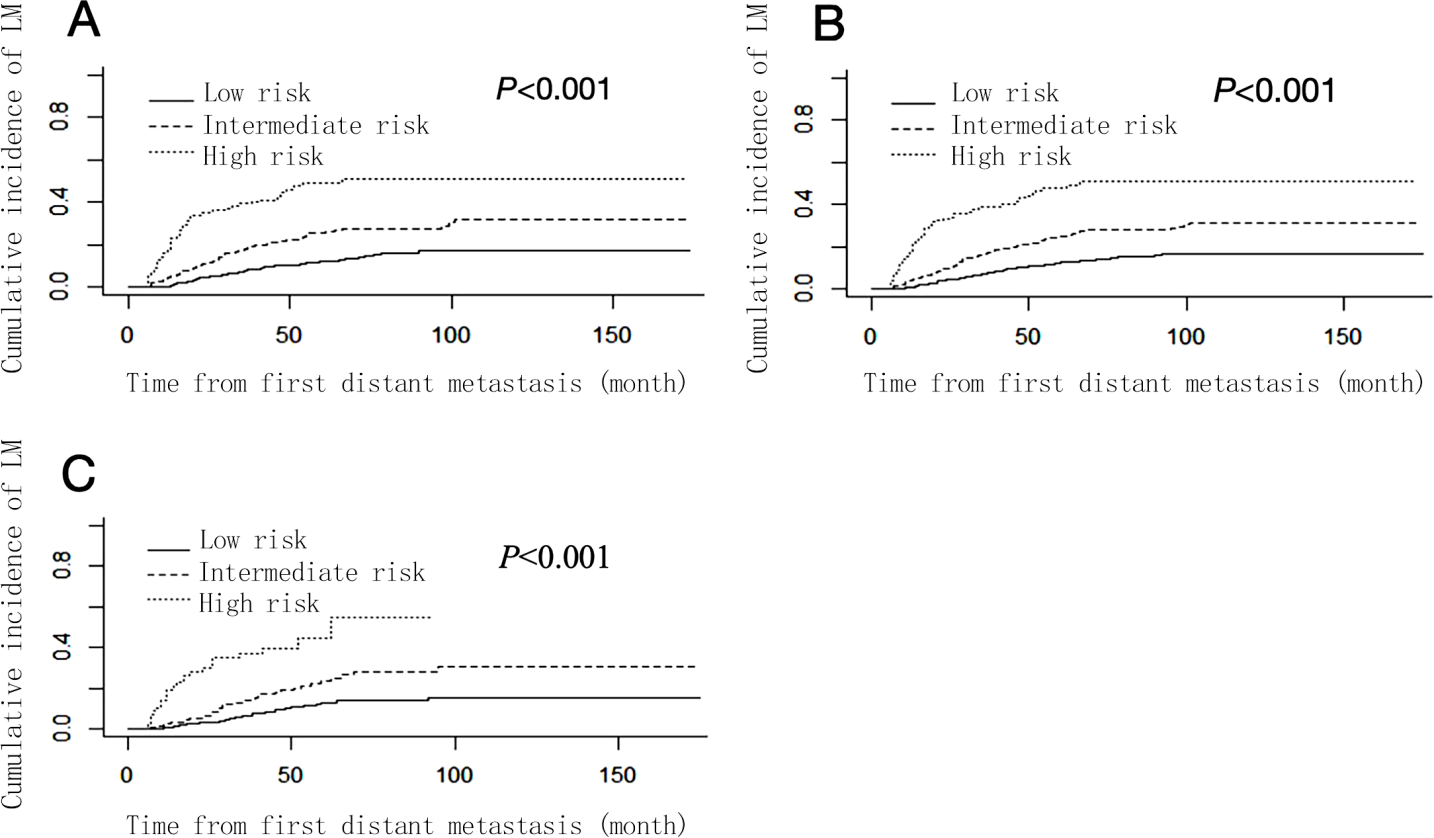
Cumulative liver metastasis incidence curves stratified by risk groups in total cohort **(A)**, training set **(B)** and validation set **(C)**.

The risk score of each patient was calculated according to the nomogram, and the X-tile software was used to divide the patients into 3 risk groups: low-risk group (793/1406, 56.40%, total score ≤ 100), intermediate-risk group (421/1406, 29.94%, 100 < total score < 234), and high-risk group (192/1406, 13.66%, total score ≥ 234). The cumulative incidence function curves (**Figure 4**) showed that the cumulative incidence of liver metastasis varied significantly among different risk groups in the total cohort, training set, and validation set (*P* < 0.05).

## 4 Discussion

The metastatic breast cancer is an incurable disease with an overall survival of only 2–3 years (22, 23). Among patients with metastatic breast cancer, patients with visceral metastasis have a worse prognosis than those without visceral metastasis. Among visceral metastasis, liver metastasis is a significant factor affecting the overall survival of metastatic breast cancer. The screening of liver metastasis from advanced breast cancer is mainly carried out by imaging techniques. In spite of their high sensitivity and specificity in diagnosing intrahepatic nodules (24–26), CT, MRI and PET-CT cannot be used as routine screening methods due to a series of problems such as radiation, high cost and contrast agent allergy. B-scan ultrasonography has become a routine screening method for liver metastasis from advanced breast cancer due to its advantages of low cost and no radiation, but its accuracy is greatly affected by subjective factors (27). Therefore, it is significantly important to construct a practical nomogram to predict the risk of subsequent liver metastasis from advanced breast cancer.

As a tool to quantify risk, nomogram is widely used in assessing the prognosis, predicting the occurrence of diseases, and assisting in making clinical decisions (28–30). Nomogram has been explored in the field of brain metastasis from metastatic breast cancer. Graesslin O (31) used logistic regression to screen out age, histological grade, molecular typing, and tumor-free survival as predictors to construct a nomogram for brain metastasis from advanced breast cancer. Lin M (20) used the Fine-Gray competing risk model to screen the risk factors of brain metastasis from triple-negative advanced breast cancer, so as to build a nomogram model to screen out patients who can benefit from brain MRI screening. However, liver metastasis is one of the poor prognostic factors in metastatic breast cancer, and there is no nomogram to predict the risk of liver metastasis from metastatic breast cancer.

We enrolled 1406 patients and identified four clinicopathological factors as predictors. A nomogram based on the Fine-Gray competing risk model was established to predict the risk of liver metastasis from metastatic breast cancer. The validation showed that this nomogram had good discrimination ability and calibration ability. To our knowledge, this study is the first to base a nomogram on the Fine-Gray competing risk model to predict the risk of liver metastasis from metastatic breast cancer.

Four independent risk factors including HER-2 status, bone metastasis, lung metastasis and menopausal status, which were closely associated with subsequent liver metastasis, were consistent with previous reports. The association between HER-2 status and liver metastasis was reported in several studies. Leone BA (32) found that Hormone receptor positive and HER2 positive was closely associated with breast cancer liver metastasis. Wei S (33) found that the liver metastasis was more likely to occur in HER2+ breast cancer patients. Moreover, there was biological evidence that could explain the association between HER2 status and liver metastasis. The CXCR4 (C-X-C chemokine receptor type 4) in breast cancer cells can promote metastasis by binding to ligands on the surface of hepatocytes or by mediation of tumor-associated macrophages, while HER2 can upregulate CXCR4 expression, which further promotes liver metastasis through the CXCL12/CXCR4 pathway (34). Bone metastasis is also included in this nomogram, and its risk score in nomogram is second only to HER-2 status. At present, there are few studies on the secondary metastases after the first bone metastasis from metastatic breast cancer. István Artúr Molnár et al. found that the liver is the most frequent site of metastases after the first bone metastasis from Hormone receptor positive breast cancer (35). About 67.00% (942/1406) of patients in this study were hormone receptor-positive. Lung metastasis was also found to be an independent predictor for subsequent liver metastasis in the present study. Studies have shown that CXCR4, which is highly expressed in breast cancer cells, is the only receptor for stromal cell-derived factor-1 (SDF-1). As a key chemokine *in vivo*, SDF-1 is highly expressed in lung and liver. This may indicate that there is a certain correlation between liver metastasis and lung metastasis in breast cancer (36–38). Therefore, metastatic breast cancer patients with first lung metastasis may also develop liver metastasis. Similarly, the menopausal status was also a predictor. Premenopausal patients are more likely to develop liver metastases than postmenopausal patients. Previous studies have shown that premenopausal breast cancer patients have a worse prognosis (39, 40) and are more prone to distant metastasis than postmenopausal patients (41).

Our nomogram can be feasibly used in clinical practice to predict liver metastasis in patients with metastatic breast cancer. We wish that it could help clinicians to identify patients with a high risk of liver metastasis. This may improve the overall survival of breast cancer patients with liver metastasis in various ways. The risk stratification system not only assists clinicians in developing more thorough follow-up plans by identifying patients with a high risk of liver metastasis and supports the early detection of asymptomatic liver metastasis so as to determine the interventions, but also it can identify high-risk groups for liver metastasis to enrich prospective clinical trial populations, so as to develop effective prevention measures against liver metastasis.

There are some limitations in this study: 1. It is a single-center retrospective study, and further prospective studies are needed to verify its accuracy for more clinical applications. 2. The risk prediction model is validated by random splitting combined with bootstrap resampling, and there is a lack of data from other centers or public databases for validation.

## 5 Conclusion

We had developed a tool that can predict subsequent liver metastasis from metastatic breast cancer, which may be useful for identifying the patients at risk of liver metastasis and guiding the individualized treatment. It had been verified that the nomogram has good discrimination and calibration, and had certain potential clinical value. This nomogram can be used to screen patients with low, intermediate and high risk of liver metastasis from metastatic breast cancer, so as to develop a more complete follow-up plan.

## Data Availability

The datasets presented in this study can be found in online repositories. The names of the repository/repositories and accession number(s) can be found in the article/**Supplementary Material**.
