# Supplementary material for: A nomogram for predicting subsequent liver metastasis in patients with metastatic breast cancer
Source: Front Oncol. 2025 Apr 16;15:1417858. doi: 10.3389/fonc.2025.1417858 (PMC12041002; doi:10.3389/fonc.2025.1417858)
Supplement: Supplementary file 1 [file Table1.docx]

Supplementary Material

**A nomogram for predicting subsequent liver metastasis in patients with metastatic breast cancer**

Xuanchen Liu^1,2^, Weipeng Zhao^1,2^, Yongsheng Jia^1^, Li Zhang^1*^, Zhongsheng Tong^1*^

^1^Department of Breast Oncology, Key Laboratory of Breast Cancer Prevention and Therapy, Tianjin’s Clinical Research Center for Cancer, National Clinical Research Center for Cancer, Tianjin Medical University Cancer Institute and Hospital, Tianjin, P.R.China

^2^Xuanchen Liu and Weipeng Zhao are the co-first authors

* Li Zhang and Zhongsheng Tong are the co-last authors

Email: Li Zhang doctor3399@163.com. Zhongsheng Tong [tongzhongsheng_on@163.com](mailto:tongzhongsheng_on@163.com)

**Table**

**Table 1.** Patients’ Baseline Demographic and Clinical Characteristics

| Characteristics | Total  (N=986)  n (%) | Training Set (N=986)  n (%) | Validation Set  (N=420)  n (%) | χ^2^ | *P* |
| --- | --- | --- | --- | --- | --- |
| Age | | | | | |
| <50 | 778 (55.33) | 551 (55.88) | 227 (54.05) | 0.401 | 0.526 |
| ≥50 | 628 (44.67) | 435 (44.12) | 193 (45.95) |  |  |
| Menopausal status | | | | | |
| Premenopause | 604 (42.96) | 423 (42.90) | 181 (43.10) | 0.005 | 0.946 |
| Postmenopause | 802 (57.04) | 563 (57.10) | 239 (56.90) |  |  |
| Histological subtype | | | | | |
| Invasive ductal | 1188 (84.50) | 836 (84.79) | 352 (83.81) | 0.215 | 0.643 |
| Others | 218 (15.50) | 150 (15.21) | 68 (16.19) |  |  |
| Histological grade | | | | | |
| I | 128 (9.10) | 91 (9.23) | 37 (8.81) | 1.009 | 0.604 |
| II | 1216 (86.49) | 855 (86.71) | 361 (85.95) |  |  |
| III | 62 (4.41) | 40 (4.06) | 22 (5.24) |  |  |
| Stage at initial diagnosis of cancer ^a^ | | | | | |
| I | 186 (13.23) | 136 (13.79) | 50 (11.90) | 2.008 | 0.571 |
| II | 565 (40.18) | 400 (40.57) | 165 (39.29) |  |  |
| III | 484 (34.42) | 336 (34.08) | 148 (35.24) |  |  |
| IV | 171 (12.17) | 114 (11.56) | 57 (13.57) |  |  |
| T stage | | | | | |
| T1 | 403 (28.66) | 295 (29.92) | 108 (25.71) | 6.209 | 0.184 |
| T2 | 747 (53.13) | 514 (52.13) | 233 (55.48) |  |  |
| T3 | 105 (7.47) | 79 (8.01) | 26 (6.19) |  |  |
| T4 | 77 (5.48) | 48 (4.87) | 29 (6.90) |  |  |
| Unknown | 74 (5.26) | 50 (5.07) | 24 (5.72) |  |  |
| N stage | | | | | |
| N0 | 433 (30.80) | 302 (30.63) | 131 (31.19) | 6.269 | 0.180 |
| N1 | 384 (27.31) | 277 (28.09) | 107 (25.48) |  |  |
| N2 | 221 (15.72) | 141 (14.30) | 80 (19.05) |  |  |
| N3 | 304 (21.62) | 222 (22.52) | 82 (19.52) |  |  |
| Unknown | 64 (4.55) | 44 (4.46) | 20 (4.76) |  |  |
| Initial surgery | | | | | |
| Complete mastectomy | 1212 (86.20) | 858 (87.02) | 354 (84.29) | 1.849 | 0.174 |
| Others | 194 (13.80) | 128 (12.98) | 66 (15.71) |  |  |
| ER status | | | | | |
| Positive | 877 (62.38) | 624 (63.29) | 253 (60.24) | 1.166 | 0.280 |
| Negative | 529 (37.62) | 362 (36.71) | 167 (39.76) |  |  |
| PR status | | | | | |
| Positive | 765 (54.41) | 553 (56.09) | 212 (50.48) | 3.736 | 0.053 |
| Negative | 641 (45.59) | 433 (43.91) | 208 (49.52) |  |  |
| HER-2 status | | | | | |
| Positive | 324 (23.04) | 226 (22.92) | 98 (23.33) | 0.028 | 0.867 |
| Negative | 1082 (76.96) | 760 (77.08) | 322 (76.67) |  |  |
| Ki-67 index (%) | | | | | |
| > 20 | 1110 (78.95) | 787 (79.82) | 323 (76.90) | 1.503 | 0.220 |
| ≤ 20 | 296 (21.05) | 199 (20.18) | 97 (23.10) |  |  |
| Initial site of MBC | | | | | |
| Lung involvement | 438 (31.15) | 302 (30.63) | 136 (32.38) | 0.422 | 0.516 |
| Bone involvement | 586 (41.68) | 402 (40.77) | 184 (43.81) | 1.119 | 0.290 |
| Lymph node involvement^c^ | 646 (45.95) | 440 (44.62) | 206 (49.05) | 2.320 | 0.128 |
| HBV infection | | | | | |
| Yes | 33 (2.35) | 22 (2.23) | 11 (2.62) | 0.193 | 0.660 |
| No | 1373 (97.65) | 964 (97.77) | 409 (97.38) |  |  |
| Fatty liver | | | | | |
| Yes | 409 (29.09) | 283 (28.70) | 126 (30.00) | 0.241 | 0.624 |
| No | 997 (70.91) | 703 (71.30) | 294 (70.00) |  |  |
| Liver cyst | | | | | |
| Yes | 82 (5.83) | 63 (6.39) | 19 (4.52) | 1.867 | 0.172 |
| No | 1324 (94.17) | 923 (93.61) | 401 (95.48) |  |  |
| Total bilirubin ^b^ | | | | | |
| ≤ 1.5ULN | 1385 (98.51) | 973 (98.68) | 412 (98.10) | 0.688 | 0.407 |
| > 1.5ULN | 21 (1.49) | 13 (1.32) | 8 (1.90) |  |  |
| ALT ^b^ | | | | | |
| ≤ 3 ULN | 1390 (98.86) | 977 (99.09) | 413 (98.33) | 1.488 | 0.223 |
| > 3 ULN | 16 (1.14) | 9 (0.91) | 7 (1.67) |  |  |
| AST ^b^ | | | | | |
| ≤ 3 ULN | 1384 (98.44) | 972 (98.58) | 412 (98.10) | 0.450 | 0.503 |
| > 3 ULN | 22 (1.56) | 14 (1.42) | 8 (1.90) |  |  |
| LDH ^b^, U/L | | | | | |
| ≤ 250 | 1002 (71.27) | 715 (72.52) | 287 (68.33) | 2.515 | 0.113 |
| > 250 | 404 (28.73) | 271 (27.48) | 133 (31.67) |  |  |
| Time between breast surgery and metastatic disease(month) | | | | | |
| >24 | 934 (66.43) | 666 (67.55) | 268 (63.81) | 2.007 | 0.367 |
| ≤24 | 301 (21.41) | 206 (20.89) | 95 (22.62) |  |  |
| Primary metastatic | 171 (12.16) | 114 (11.56) | 57 (13.57) |  |  |

a: Based on the American Joint Committee on Cancer Staging Manual, 8th ed.

b: All variables were measured within 2 weeks preceding the first-line treatment of metastatic disease.

c:It includes the lymph nodes of secondary relapse after surgery and the first metastasis site of de novo metastatic breast cancer

**Table 2.** Univariable and Multivariable Competing-risk Regression Analysis for Liver Metastasis-free Survival

| Characteristics | Univariable Analysis | | |  | Multivariable Analysis | | |
| --- | --- | --- | --- | --- | --- | --- | --- |
|  | SHR | 95% CI | *P* |  | SHR | 95% CI | *P* |
| Age | | | | | | | |
| ≥50 | 1 |  |  |  | 1 |  |  |
| <50 | 1.36 | (1.03–1.79) | 0.030* |  | 1.09 | (0.80–1.50) | 0.580 |
| Menopausal status | | | | | | | |
| Postmenopausal | 1 |  |  |  | 1 |  |  |
| Premenopausal | 1.58 | (1.21–2.06) | <0.001* |  | 1.52 | (1.12–2.06) | 0.007* |
| Histological subtype | | | | | | | |
| Invasive ductal | 1 |  |  |  |  |  |  |
| Others | 0.98 | (0.68–1.40) | 0.890 |  |  |  |  |
| Histological grade | | | | | | | |
| I | 1 |  |  |  |  |  |  |
| II | 1.06 | (0.80–1.46) | 0.641 |  |  |  |  |
| III | 1.52 | (0.36–6.19) | 0.571 |  |  |  |  |
| Stage at initial diagnosis of cancer ^a^ | | | | | | | |
| I | 1 |  |  |  |  |  |  |
| II | 1.16 | (0.79–1.70) | 0.860 |  |  |  |  |
| III | 1.12 | (0.76–1.65) | 0.895 |  |  |  |  |
| IV | 1.54 | (0.90–2.64) | 0.649 |  |  |  |  |
| T stage | | | | | | | |
| T1 | 1 |  |  |  |  |  |  |
| T2 | 1.13 | (0.85–1.50) | 0.420 |  |  |  |  |
| T3 | 1.07 | (0.66–1.72) | 0.790 |  |  |  |  |
| T4 | 2.07 | (0.93–4.65) | 0.070 |  |  |  |  |
| N stage | | | | | | | |
| N0 | 1 |  |  |  |  |  |  |
| N1 | 1.14 | (0.81–1.60) | 0.460 |  |  |  |  |
| N2 | 1.03 | (0.68–1.57) | 0.870 |  |  |  |  |
| N3 | 1.06 | (0.75–1.51) | 0.740 |  |  |  |  |
| Initial surgery | | | | | | | |
| Complete mastectomy | 1 |  |  |  |  |  |  |
| Others | 1.22 | (0.79–1.86) | 0.360 |  |  |  |  |
| ER status | | | | | | | |
| Positive | 1 |  |  |  | 1 |  |  |
| Negative | 1.76 | (1.29–2.38) | <0.001* |  | 1.37 | (0.94–2.01) | 0.100 |
| PR status | | | | | | | |
| Positive | 1 |  |  |  | 1 |  |  |
| Negative | 1.70 | (1.28–2.27) | <0.001* |  | 1.30 | (0.91–1.86) | 0.160 |
| HER-2 status | | | | | | | |
| Negative | 1 |  |  |  |  |  |  |
| Positive | 2.29 | (1.72–3.04) | <0.001* |  | 2.28 | (1.71–3.03) | <0.001* |
| Ki-67 index (%) | | | | | | | |
| > 20 | 1 |  |  |  |  |  |  |
| ≤20 | 0.89 | (0.64–1.24) | 0.500 |  |  |  |  |
| Initial site of MBC | | | | | | | |
| Lung involvement(vs.no) | 2.05 | (1.56–2.68) | <0.001* |  | 2.01 | (1.53–2.65) | <0.001* |
| Bone involvement(vs.no) | 2.16 | (1.65–2.83) | <0.001* |  | 1.89 | (1.43–2.50) | <0.001* |
| Lymph node involvement(vs.no) | 1.19 | (0.91–1.56) | 0.220 |  |  |  |  |
| HBV infection | | | | | | | |
| No | 1 |  |  |  |  |  |  |
| Yes | 1.69 | (0.54–5.33) | 0.370 |  |  |  |  |
| Fatty liver | | | | | | | |
| Yes |  |  |  |  |  |  |  |
| No | 1.07 | (0.79–1.45) | 0.650 |  |  |  |  |
| Liver cyst | | | | | | | |
| No | 1 |  |  |  |  |  |  |
| Yes | 1.10 | (0.62–1.96) | 0.740 |  |  |  |  |
| Total bilirubin ^b^ | | | | | | | |
| ≤1.5ULN | 1 |  |  |  |  |  |  |
| >1.5ULN | 2.93 | (0.40–21.50) | 0.290 |  |  |  |  |
| ALT ^b^ | | | | | | | |
| ≤3 ULN | 1 |  |  |  |  |  |  |
| >3 ULN | 2.02 | (0.27–15.00) | 0.490 |  |  |  |  |
| AST ^b^ | | | | | | | |
| ≤3 ULN | 1 |  |  |  |  |  |  |
| >3 ULN | 1.51 | (0.37–6.14) | 0.560 |  |  |  |  |
| LDH ^b^, U/L | | | | | | | |
| ≤250 | 1 |  |  |  |  |  |  |
| >250 | 1.14 | (0.85–1.52) | 0.390 |  |  |  |  |
| Time between breast surgery and metastatic disease(month) | | | | | | | |
| >24 | 1 |  |  |  |  |  |  |
| ≤24 | 0.87 | (0.65~1.17) | 0.350 |  |  |  |  |
| Primary metastatic | 0.83 | (0.50～1.39) | 0.481 |  |  |  |  |

a: Based on the American Joint Committee on Cancer Staging Manual, 8th ed.

b: All variables were measured within 2 weeks preceding the first-line treatment of metastatic disease.

A

**Table 3.** Scores of Clinical Factors in Each Subgroup

| Variables | Score (Point) |
| --- | --- |
| HER-2 status |  |
| Negative | 0 |
| Positive | 100 |
| Bone metastasis |  |
| No | 0 |
| Yes | 94 |
| Lung metastasis |  |
| No | 0 |
| Yes | 82 |
| Menopausal status |  |
| Postmenopausal | 0 |
| Premenopausal | 58 |
